# Overexpression of WDR79 in non‐small cell lung cancer is linked to tumour progression

**DOI:** 10.1111/jcmm.12759

**Published:** 2016-02-05

**Authors:** Yang Sun, Chao Yang, Jieying Chen, Xin Song, Zhen Li, Minlan Duan, Jianglin Li, Xiaoxiao Hu, Kuangpei Wu, Guobei Yan, Cai Yang, Jing Liu, Weihong Tan, Mao Ye

**Affiliations:** ^1^ Molecular Science and Biomedicine Laboratory State Key Laboratory for Chemo/Biosensing and Chemometrics College of Biology College of Chemistry and Chemical Engineering Collaborative Innovation Center for Molecular Engineering for Theranostics Hunan University Changsha Hunan China; ^2^ College of Life and Environmental Sciences Gannan Normal University Ganzhou Jiangxi China; ^3^ Cancer Biotherapy Center Tumor Hospital of Yunnan Province Affiliated with Kunming Medical University Kunming Yunnan China; ^4^ State Key Laboratory of Medical Genetics & School of Life Sciences Central South University Changsha Hunan China

**Keywords:** lung cancer, cell cycle, apoptosis, proliferation

## Abstract

WD‐repeat protein 79 (WDR79), a member of the WD‐repeat protein family, acts as a scaffold protein, participating in telomerase assembly, Cajal body formation and DNA double‐strand break repair. Here, we first report that WDR79 is frequently overexpressed in cell lines and tissues derived from non‐small cell lung cancer (NSCLC). Knockdown of WDR79 significantly inhibited the proliferation of NSCLC cells *in vitro* and *in vivo* by inducing cell cycle arrest and apoptosis. WD‐repeat protein 79 ‐induced cell cycle arrest at the G0/G1 phase was associated with the expression of G0/G1‐related cyclins and cyclin‐dependent kinase complexes. We also provide evidence that WDR79 knockdown induces apoptosis *via* a mitochondrial pathway. Collectively, these results suggest that WDR79 is involved in the tumorigenesis of NSCLC and is a potential novel diagnostic marker and therapeutic target for NSCLC.

## Introduction

Lung cancer is the most commonly diagnosed malignant tumour and remains the leading cause of cancer‐related deaths worldwide 1. Non‐small cell lung cancer (NSCLC) and small cell lung cancer (SCLC) are the two major pathological types of lung cancer, which can be morphologically differentiated under the microscope. Non‐small cell lung cancer is the most commonly diagnosed lung cancer and accounts for approximately 85% of all cases. Despite advances in surgery, radiation therapy and chemotherapy, survival rates remain discouragingly low, with a 5‐year survival rate of only 15% 2. Therefore, the identification of new molecular targets involved in the pathogenesis of NSCLC could play a key role in developing novel therapeutic strategies to treat this malignancy.

WD‐repeat domains bear a C‐terminal tryptophan‐aspartic acid dipeptide and fold into β‐propellers that mediate protein complex formation 3. The human genome encodes approximately 300 such proteins engaged in various cellular activities, that is, chromatin assembly, gene transcription, RNA metabolism, vesicular trafficking, cytoskeleton remodelling, signalling, apoptosis and cell cycle regulation 4. With recent developments in research, an increasing number of WD‐repeat proteins have been found to be involved in tumorigenesis, and some, such as Rack1 5, WDRPUH 6, Endonuclein 7 and STRAP 8, function in tumour promotion. Other WD‐repeat proteins are tumour suppressors, including FBW7 9 and WDR6 10.

WD‐repeat protein 79 (WDR79) (also referred to as WRAP53/TCAB1) belongs to the WD‐repeat protein family. WD‐repeat protein 79 contains six individual WD‐repeat domains, and its homologues share high sequence identities from yeast to humans. Studies have revealed that WDR79 acts as a scaffold protein that participates in telomerase assembly, Cajal body formation and DNA double‐strand break repair 11, 12, 13. In addition to the WDR79 protein, the *WDR79* gene on chromosome 17p13 encodes an antisense transcript for p53 stabilization (referred to as WRAP53α) that is produced from an alternative transcription start site 14. Despite this connection to p53, neither WDR79 (WRAP53β/TCAB1) transcripts nor proteins are involved in the regulation of p53 15.

WD‐repeat protein 79 has been implicated in human disease. Germline mutations in WDR79 that affect the WD‐repeat domain result in dyskeratosis congenita 16. Overexpression of WDR79 has been observed in primary nasopharyngeal carcinoma 17, oesophageal squamous cell carcinoma 18 and rectal cancer 19. Moreover, single nucleotide polymorphisms in the WDR79 gene have been linked to an increased risk of ER‐negative breast cancer 20 and ovarian cancer 21, 22.

In this study, we establish a link between WDR79 expression and NSCLC progression by investigating the functional role of WDR79 in NSCLC tumorigenesis *in vitro* and *in vivo*. We provide evidence that WDR79 is involved in cell cycle regulation by affecting the expression of G0/G1‐related cyclins and cyclin‐dependent kinase (CDK) complexes. We also show that WDR79 mediates the induction of apoptosis *via* mitochondrial pathways. Collectively, our results suggest that WDR79 is a potential novel diagnostic marker and therapeutic target for NSCLC.

## Materials and methods

### Lung cancer tissue samples and cell lines

Fifty lung cancer tissues and 44 adjacent normal tissues were obtained from lung cancer patients. Fresh specimens were stored at −80°C after being snap‐frozen in liquid nitrogen until analysis. Human NSCLC cell lines A549, H1299 95‐C, 95‐D, and HTB182, as well as normal lung epithelial cell line HBE, were maintained in RPMI‐1640 (Gibco BRL Co. Ltd., Grand Island, NY, USA) medium supplemented with 10% foetal bovine serum (Gibco BRL Co. Ltd.) at 37°C in 5% CO_2_ humidified incubators.

### Immunohistochemistry

Formalin‐fixed, paraffin‐embedded samples were sectioned at 5 μM. Sections were treated with antigen retrieval buffer. Specifically, WDR79 antibody (Bethyl Laboratories, Inc., Montgomery, TX, USA) was applied overnight at a dilution of 1:100. After PBS washing, 50 μl biotinylated secondary antibody was incubate with sections and then conjugate with 20 μl streptavidin‐peroxidase for 30 min. Colour visualization was achieved by incubating the sections with 3,3′‐diaminobenzidine (Dako Corporation, Carpinteria, CA, USA) for 5 min. and counterstained with haematoxylin. WD‐repeat protein 79 expression in lung cancer tissues and corresponding normal lung tissue specimens from NSCLC patients were reviewed and scored under a light microscope by two independent pathologists (Song X and Li Z) who were not aware of the clinicopathological data. In the event of a discrepancy, a consensus interpretation was reached under a two‐headed microscope. As WDR79 is mainly located in the nuclei, nuclear staining of ≥10% of the cancer cells was considered positive. If fewer than 10% of cancer cells nuclear were stained, the slides were scored as negative WDR79 expression.

### RNA interference

WDR79 siRNA sequence (sense sequence 5′‐AATCAGCGCATCTACTTCGAT‐3′, antisense sequence 5′‐AAATCGAAGTAGATGCGCTGA‐3′), which had been proved to knock down WDR79 effectively, were purchased from GenePharma (Shanghai, China) 14. To stably knockdown endogenous WDR79 in some case, we used lentivirus‐packaging shRNA expression vector (purchased from GenePharma) to infect cells. WDR79 shRNA target sequences were 5′‐AATCAGCGCATCTACTTCGAT‐3′. The control shRNA sequence was 5′‐TTCTCCGAACGTGTCACGTTTC‐3′.

### Western blot

Whole cell extractions were generated using M‐PER lysis buffer (Pierce, Rockford, IL, USA) from lung cancer cells, and protein concentrations were determined by BCA protein assay kit (Pierce). Standard Western blotting was performed with the routine approach. Primary WDR79 antibody (Bethyl Laboratories, Inc.) was diluted at 1:2000 in 5% blocking milk (Bio‐Rad, Hercules, CA, USA). Bax, Bcl‐2, Cyclin D1, cyclin E, pRb, PRAP, Bcl‐2, CYCs (Sangon Biotechnology, Shanghai, China) antibodies were diluted at 1:500. Caspase3 (Santa Cruz Biotechnology, Santa Cruz, CA, USA) were diluted at 1:1000. GAPDH 1:10,000 (KangChen Bio‐tech Inc., Shanghai, China) was used as an internal control.

### Immunofluorescence assay

Cells were fixed with 4% paraformaldehyde for 30 min. and permeabilized with 0.2% TritonX‐100 for 15 min., blocked with 5% bovine serum albumin and incubated with anti‐WDR79 (Bethyl Laboratories, Inc.) or anti‐CYCs (Sangon Biotechnology) antibodies at 4°C overnight, followed by a dylight 594‐conjugated goat anti‐rabbit IgG antibody and dylight 488‐conjugated antimouse IgG antibody (ImmunoReagents, Inc., Raleigh, NC, USA). Cells were stained with 2‐(4‐Amidinophenyl)‐6‐indolecarbamidine dihydrochloride (DAPI) (Beyotime Biotechnology, Haimen, China) for 10 min., and the images were acquired with a confocal microscope.

### Cell proliferation assay

After WDR79 overexpression or knockdown for 24 hrs, cells were seed in 96‐well plates at a density of 10^3^ cells per well. At the indicated time‐points, the 3‐(4, 5‐dimethylthiazol‐2‐yl)‐2, 5‐diphenyltetrazolium bromide (Sigma‐Aldrich, St. Louis, MO, USA) solution was added to each well and incubated at 37°C for another 4 hrs. The supernatants were then aspirated carefully, and the formazan product was dissolved with 100 μl dimethyl sulfoxide. The absorbance was measured at a wavelength of 570 nm with a microplate reader (Bio‐Tek, Doraville, GA, USA).

### Colony formation assay

WD‐repeat protein 79 was overexpressed or knocked down for 24 hrs and seeded in 6‐well plates at a density of 10^4^ cells per well. Cells were selected with G418 or puromycin for 2 weeks, fixed with 4% paraformaldehyde for 30 min. and stained with 1% crystal violet for 10 min. The colony numbers were counted with Image J software.

### Morphological analysis of nuclei

Apoptotic morphological changes in the nuclear chromatin of cells were detected by Hoechst 33258 staining. A549 and H1299 cells were seeded at a density of 100,000 cells in 35‐mm culture plates, allowed to recover overnight and then treated with WDR79 siRNA for 48 hrs. Following treatment, treated cells were fixed for 30 min. at room temperature. Hoechst 33258 was added to the cells, which were incubated for 10 min. at room temperature and washed with PBS twice.

### Cell cycle

Cells were collected and fixed with 70% cold ethanol at −20°C for 48 hrs. The monodispersed cells were incubated with propidium iodide (PI) (Beyotime Institute of Biotechnology) at 37°C for 30 min. Cell cycle assay was performed in a flow cytometer (BD FACSVerse^™^, BD BioSciences, Franklin Lakes, NJ, USA).

### Apoptosis assay

Apoptosis was assessed by Annexin V/PI double staining assay using Annexin V‐FITC Apoptosis Detection kits (Beyotime Institute of Biotechnology, Haimen, China). Cells transfected with siRNA were collected and resuspended in Annexin V‐PI binding buffer. The cells were then stained according to the manufacturer's instructions. The stained cells were analysed by flow cytometry (BD FACSVerse^™^, BD BioSciences, Franklin Lakes, NJ, USA).

### Caspase activity assay

Cells were treated with WDR79 siRNA or control siRNA for 48 hrs and then lysed with lysis buffer on ice for 15 min. The lysates were centrifuged at 18407 *g* at 4°C for 15 min. The supernatants were collected, and protein concentration was determined by BCA (bicinchoninic acid) protein assay (Pierce). Cellular extracts (100 μg) were then incubated in a 96‐well plate with 20 ng Ac‐DEVD‐pNA (caspase‐3 activity), Ac‐IETD‐pNA (caspase‐8 activity) or Ac‐LEHD‐pNA (caspase‐9 activity) (Beyotime Institute of Biotechnology) at 37°C for 6 hrs. Caspase activity was detected by cleavage of the Ac‐DEVD‐pNA or Ac‐IEVD‐pNA or Ac‐LEHD‐pNA substrate to pNA, which was measured at 405 nm using a microplate reader (Bio‐Tek).

### *In vivo* tumour formation assay

To establish lung cancer xenografts in nude mice, a total of 5 × 10^6^ A549 cells in log phase stably transfected with either control or WDR79 targeting shRNA vectors were harvested, washed twice with DPBS, suspended in 100 μl DPBS and injected into the right flank site of each mouse (*n* = 5 for each group). All the mice were kept in pathogen‐free environments, and the xenografts were evaluated using a calliper every 2 days for 1 month. Tumour volume was calculated according to the following formula: V = 0.5 (length × width^2^). All mice were killed at day 35.

### Statistical analysis

Statistical Package for Social Science (SPSS) version 19.0 for windows (SPSS Inc., Chicago, IL, USA) and GraphPad Prism 6 (GraphPad Software Inc., San Diego, CA, USA) were used to analyse the data. Student's *t*‐test was used to compare the data between every two groups respectively. For all statistical analysis, *P*‐value less than 0.05 was considered statistically significant.

## Results

### WDR79 is overexpressed in human NSCLC tissues and cell lines

**Figure 1:**
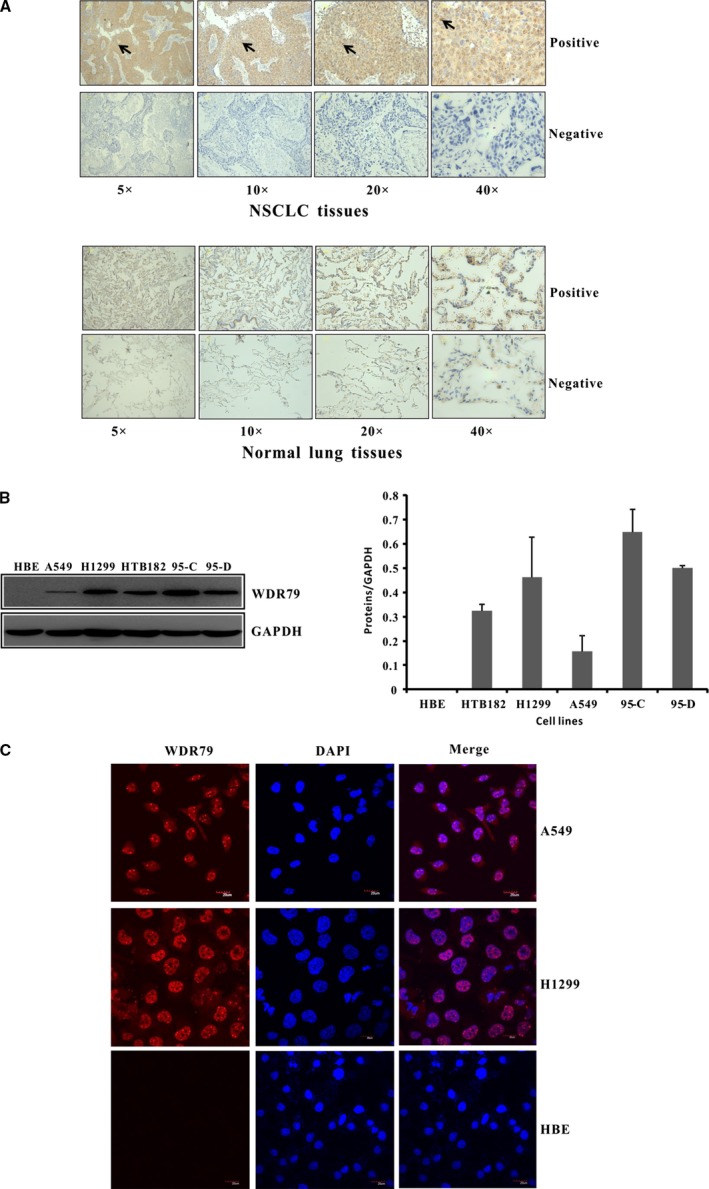
The expression and localization of WDR79 protein in NSCLC. (**A**) Representative IHC staining results for WDR79 in human NSCLC tissues and corresponding normal lung tissues are shown. (**B**) The expression level of WDR79 protein in HBE, HT182, H1299, A549, 95‐C and 95‐D cells was examined by Western blotting using WDR79 antibody. (**C**) A549 and H1299 cells were fixed and incubated with WDR79 antibody, followed by staining with DyLight594‐conjugated IgG for WDR79 and DAPI staining for nuclei identification.

Because WDR79 is an essential component of telomerase, which is highly activated in most lung cancers 23, we first analysed the protein expression of WDR79 using immunohistochemistry in 50 NSCLC tissues and 44 normal lung tissues. WD‐repeat protein 79 was predominantly localized in the nucleus, however, cytoplasmic staining was also partially present (Fig. 1A). Overall, 64% of NSCLC tissues were positive for WDR79 expression, and 36% were negative. In contrast, 43.2% of normal lung tissue samples were positive for WDR79 expression, and 56.8% were negative (Table 1). Representative positive and negative stains for WDR79 in NSCLC tissue and normal lung tissue are shown in Figure 1A. Analysis of the staining data revealed that NSCLC tissues had significantly higher positive WDR79 expression than normal lung tissues (*P* < 0.05; Table 1). However, no correlations were observed between WDR79 expression and age, sex or tumour differentiation.

**Table 1:** Expression of STIP in lung cancer tissue and corresponding normal lung tissue

Characteristic	No. of samples	No. of positive samples (%)	Statistical significance^a^
NSCLC tissues	50	32 (64.0)	*P* = 0.043
Normal lung tissues	44	19 (43.2)	

a Chi‐squared tests.

We further characterized WDR79 expression in different lung cell lines. A high level of WDR79 expression was observed in all NSCLC cell lines, including giant cell lung carcinoma 95‐C and 95‐D cells, squamous cell lung carcinoma HTB182 cells and lung adenocarcinoma A549 and H1299 cells (Fig. 1B). However, WDR79 expression was not detected in normal lung epithelial HBE cells, which showed significantly different expression when compared with NSCLC cells. We also checked the mRNA level of WDR79 in HBE cell line and found that WDR79 can be transcript (Fig. S1A). We speculated that it was possible that the translation of WDR79 mRNA to protein might be terminated or WDR79 protein might be degraded in HBE cells. Because subcellular localization is a key index of function, the distribution of endogenous WDR79 was further verified in NSCLC cells by indirect immunofluorescence staining. We found that WDR79 was primarily localized in the nucleus of A549 and H1299 cells. Consistent with previous studies, a portion of the nuclear WDR79 protein fraction was enriched in nuclear Cajal bodies (Fig. 1C). In contrast, endogenous WDR79 was absent in normal lung epithelial HBE cells (Fig. 1C). These data show that WDR79 is overexpressed in NSCLC tissues and cells and it might, therefore, play an important role in the tumorigenesis of NSCLC.

### WDR79 promotes NSCLC cell proliferation

**Figure 2:**
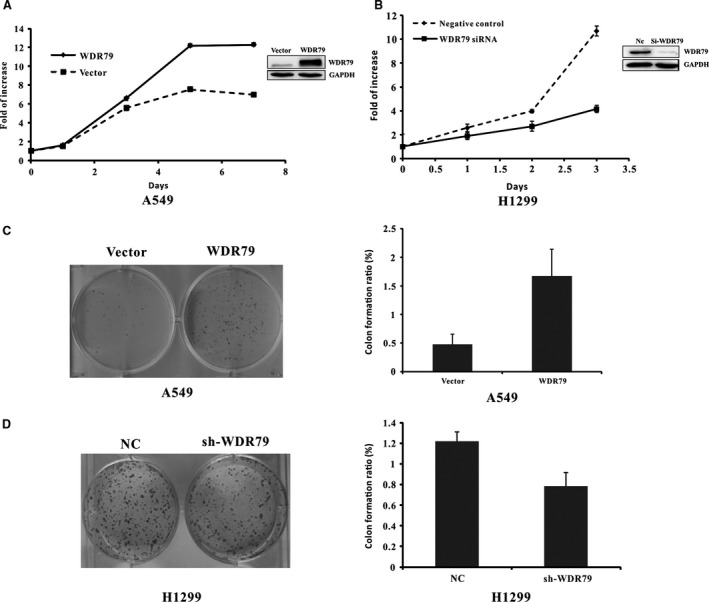
WDR79 is involved in the proliferation of NSCLC cells. (**A** and **B**) After A549 cells were transfected with WDR79 plasmid or empty vector (**A**) or H1299 cells were transfected with WDR79 siRNA or control siRNA (**B**), cell viability was determined *via* the MTT assay at the indicated time‐points. (**C** and **D**) After A549 cells were transfected with WDR79 plasmid or empty vector (**C**) or H1299 cells were infected with WDR79 shRNA or control shRNA (**D**), cells were selected in the presence of 1 mg/ml puromycin for 10 days. Colonies were stained with 0.1% crystal violet and subsequently photographed (left) and counted (right).

Endogenous WDR79 protein showed different expression levels in all NSCLC cell lines tested. Moreover, WDR79 expression was lower in A549 cells than in NSCLC cells. Interestingly, we found that H1299 cells, which express higher levels of WDR79, showed more rapid proliferation than A549 cells (Fig. S1B). Meanwhile, the normal lung epithelial HBE cells in which WDR79 were not detected showed the lowest proliferation compared with A549 and H1299 cells (Fig. S1C). To further establish a link between WDR79 expression and NSCLC cell proliferation, loss‐of‐function and gain‐of‐function studies were performed. When plasmids expressing Flag‐WDR79 were transfected into A549 cells, we found that WDR79 enhanced the proliferation of A549 cells compared with control cells transfected with empty vector (Fig. 2A). In contrast, down‐regulation of endogenous WDR79 using siRNA inhibited the proliferation of H1299 cells (Fig. 2B). Furthermore, colony formation assays indicated that the number of colonies significantly increased by approximately threefold in A549 cells as a result of WDR79 overexpression and decreased by ~1.5‐fold in H1299 cells as a result of WDR79‐knockdown. These results indicate that WDR79 promotes NSCLC cell proliferation.

### Knockdown of WDR79 causes cell cycle arrest at the G0/G1 phase by regulating G0/G1‐related regulatory proteins

**Figure 3:**
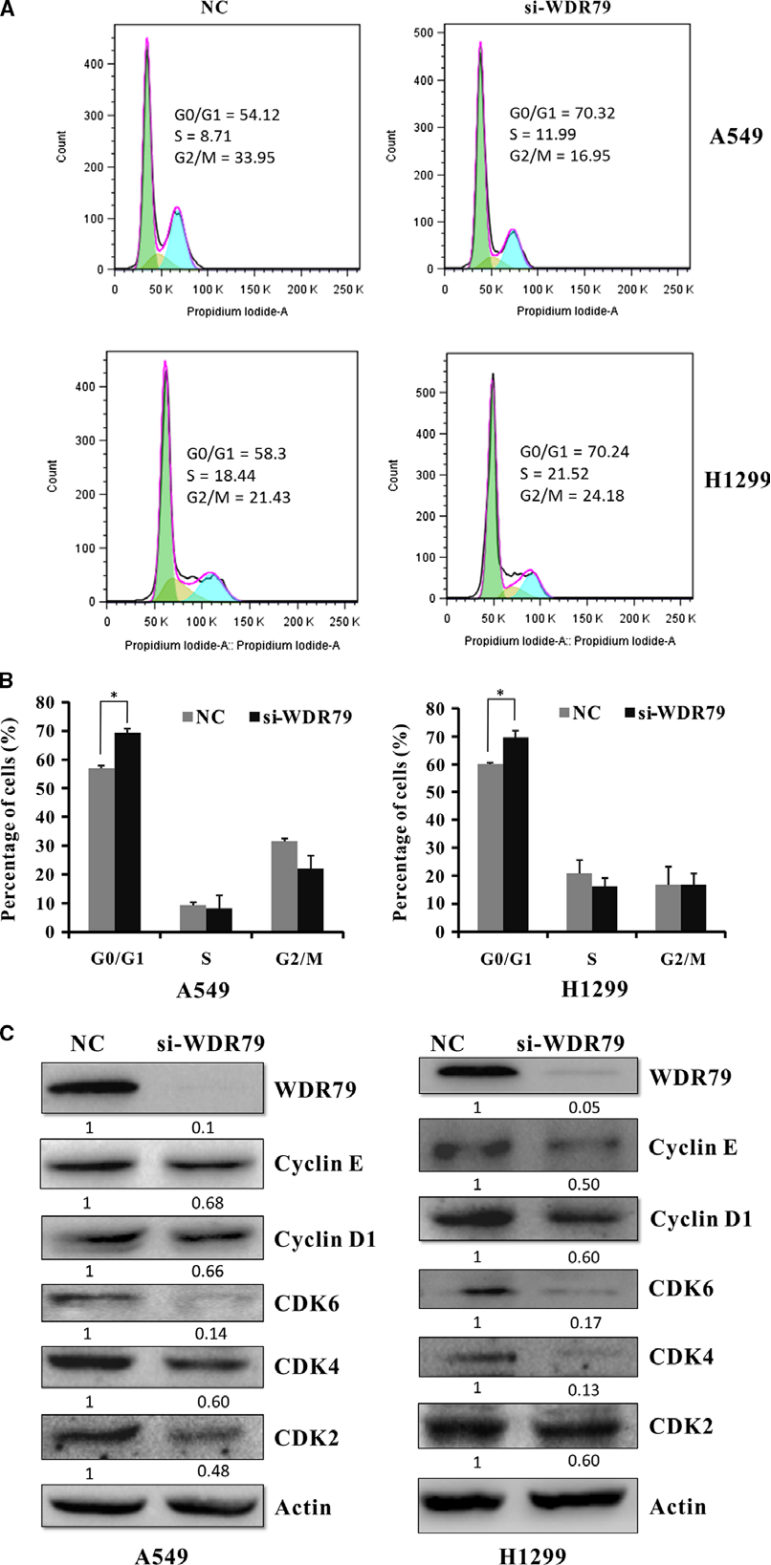
Knockdown of WDR79 causes cell cycle arrest at the G0/G1 phase. (**A**) After A549 and H1299 cells were transfected with WDR79 siRNA or control siRNA, cells were subjected to cell cycle analysis. A typical cell cycle distribution is shown. (**B**) The percentages of A549 and H1299 cells in the different cell cycle phases are shown. (**C**) The expression of G0/G1‐related cyclins and CDKs were examined by Western blotting using the indicated antibodies.

Cell proliferation depends on cell cycle progression. To examine whether the effect of WDR79 on NSCLC cell proliferation was mediated *via* cell cycle regulation, we first used flow cytometry to examine the cell cycle distribution of NSCLC cells. Following WDR79 knockdown in H11299 and A549 cells using WDR79 siRNA, the cells were harvested and stained with PI for flow cytometric analysis. Compared with the control cells, the percentage of cells in the G0/G1 phase significantly increased from 59.91 ± 0.69% to 69.57 ± 2.4% and from 56.98 ± 2.61% to 69.33 ± 1.39% in WDR79‐silenced H1299 and A549 cells respectively (Fig. 3A and B). Similar results were also obtained in A549 and H1299 cells using WDR79 shRNA to silence WDR79 expression (Fig. S2A and B). In contrast, overexpressed WDR79 in A549 cells caused a decrease in cells in the G0/G1 phase from 70.7 ± 1.33% to 63.7 ± 1.06% (Fig. S2C).

To investigate the mechanism underlying the induction of cell cycle arrest, we tested the effect of WDR79 knockdown on G0/G1‐associated cyclins and CDKs. As shown in Figure 3C, the knockdown of WDR79 decreased the protein levels of cyclin D1, cyclin E, CDK6, CDK4 and CDK2 in both A549 and H1299 cells. Taken together, these results indicate that the inhibition of WDR79 expression induces cell cycle arrest at the G0/G1 phase by regulating the expression of G0/G1‐associated cyclins and CDKs.

### Depletion of WDR79 induces apoptosis

**Figure 4:**
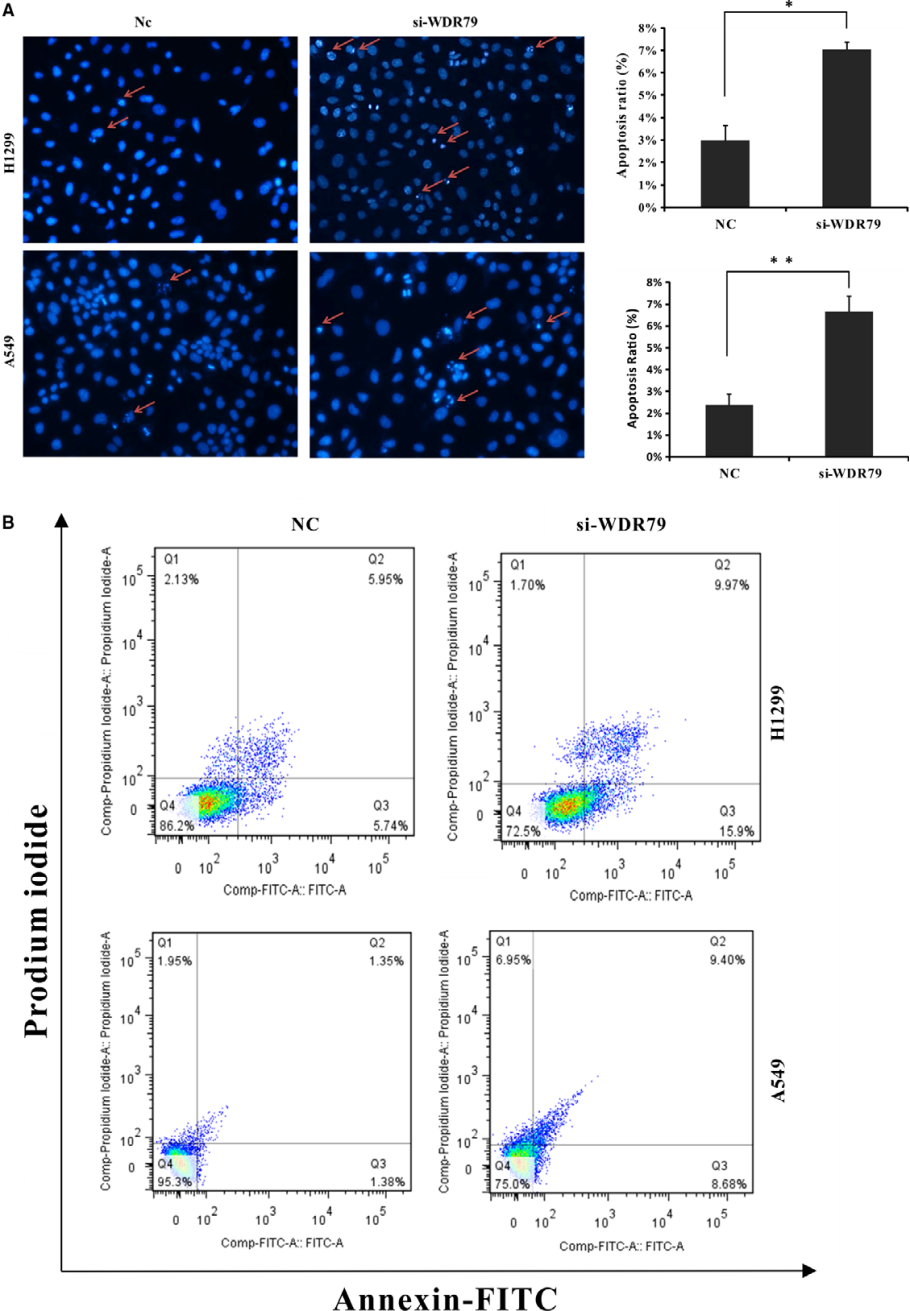
The depletion of WDR79 induces apoptosis. (**A**) Apoptotic changes in nuclear morphology were observed *via* Hoechst 33258 staining by fluorescence microscopy. The percentage of apoptotic cells is presented as the mean S.D. (*n* = 3; *P < 0.05; **P < 0.01). (**B**) A549 and H1299 cells were transfected with WDR79 siRNA or control siRNA and then stained with Annexin V‐FITC/PI. The percentage of apoptotic cells was analysed *via* flow cytometry.

To determine whether reduced cell numbers following WDR79 knockdown were related to the induction of apoptosis, nuclear morphological changes were investigated in WDR79‐silenced H1299 and A549 cells by staining with the fluorochrome Hoechst 33258. Based on chromatin condensation and nuclear fragmentation, a significant increase in apoptotic nuclei was observed in the WDR79 siRNA‐treated cells compared with the control‐treated cells (Fig. 4A). Furthermore, apoptotic cells were analysed by flow cytometry using FITC‐conjugated Annexin V and PI double staining. Compared with the control cells, knockdown of WDR79 in the H1299 and A549 cells resulted in a significant increase in the percentage of early apoptotic cells (Annexin V‐positive, PI‐negative) from 5.74% to 15.9% and from 1.36% to 8.58% respectively (Fig. 4B). These findings indicate that WDR79 knockdown induces the apoptotic death of NSCLC cells.

### Mitochondrial pathways are involved in WDR79 depletion‐induced apoptosis

**Figure 5:**
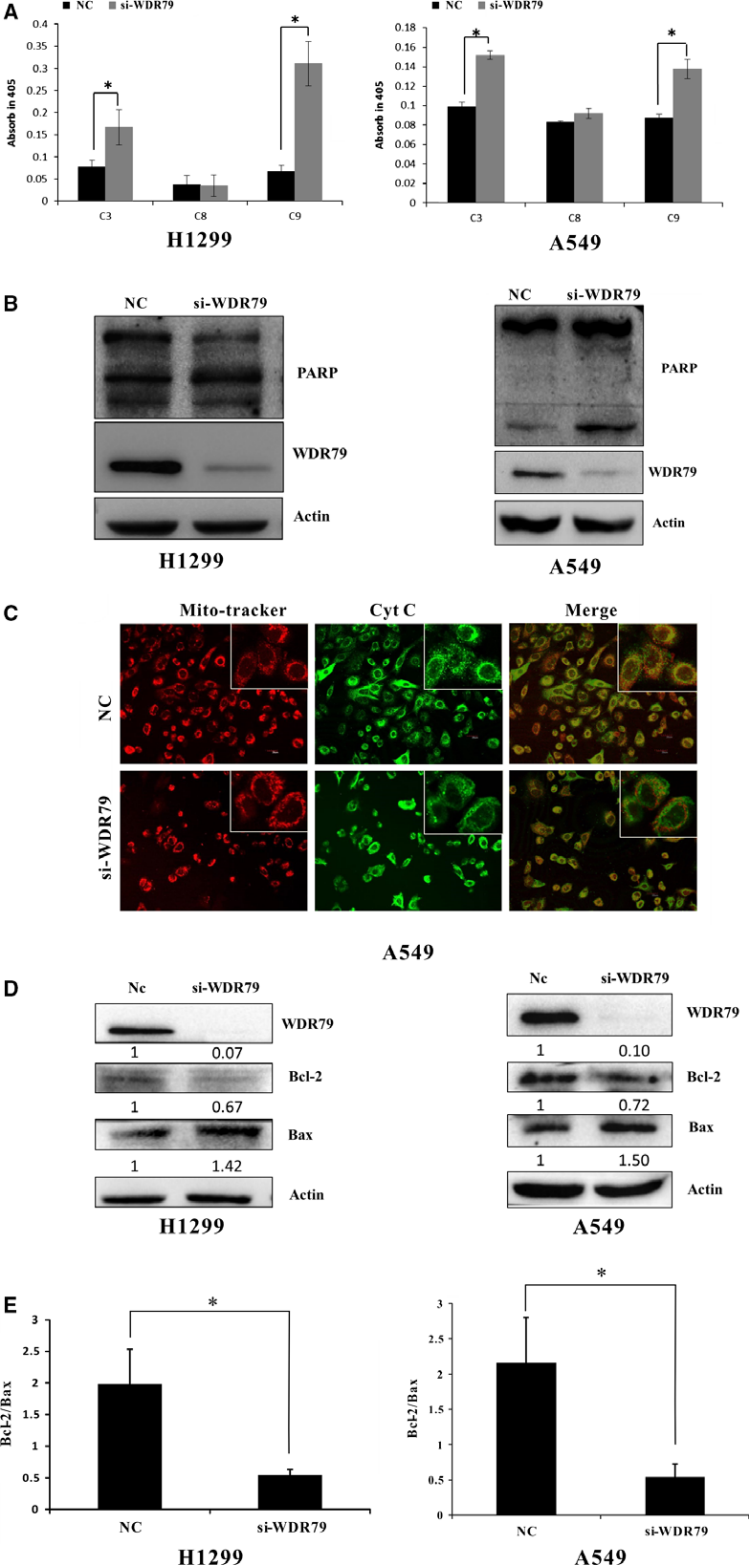
The mitochondrial pathway is involved in WDR79‐mediated apoptosis. (**A**) After A549 and H1299 cells were transfected with control siRNA or WDR79 siRNA for 72 hrs, caspase‐3, caspase‐8 and caspase‐9 activities were detected and presented as fold‐change relative to that of control cells, *P < 0.05. (**B**) A549 and H1299 cells were transfected with control siRNA or WDR79 siRNA. The expression levels of PARP were examined by Western blot using the indicated antibodies. (**C**) After A549 cells were transfected with WDR79 siRNA or control siRNA, cells were fixed and then incubated with cytochrome *c* antibody, followed by staining with DyLight488‐conjugated IgG for cytochrome *c* and Mitotracker for mitochondria. (**D** and **E**) After Cells were transfected with WDR79 siRNA or control siRNA, total cellular protein extracts were prepared, and the protein expression of Bcl‐2 and Bax was analysed *via* Western blot. Actin was used as an internal control (**D**). The Bax/Bcl‐2 expression ratio was quantified *via* densitometry (**E**), *P < 0.05.

To reveal the underlying mechanism of WDR79 depletion‐induced apoptosis, caspase activation was analysed in H1299 and A549 cells. As shown in Figure 5A, the activity levels of caspase‐3 and caspase‐9 were significantly elevated in WDR79 siRNA‐treated cells compared with negative control siRNA‐treated cells, whereas caspase‐8 activity was not affected (Fig. 5A). The activation of caspases results in the proteolytic cleavage of numerous cellular substrates. Among these, poly (ADP‐ribose) polymerase (PARP) is a substrate for caspase‐3 cleavage during apoptosis. To further verify the activation of caspase‐3, the cleavage of PARP was measured in H1299 and A549 cells. Compared with the negative control cells, WDR79‐depleted cells displayed dramatically higher levels of cleaved PARP, suggesting that the depletion of WDR79 resulted in caspase‐3‐dependent apoptosis (Fig. 5B). Because the release of cytochrome *c* from mitochondria is required for the activation of caspase‐9, we next examined the subcellular distribution of cytochrome *c*. When A549 cells were labelled with Mitotracker, which labels mitochondria, and FITC‐conjugated antibody against cytochrome *c*, we observed an intense green colour in the merged images of cells transfected with WDR79 siRNA relative to the control cells. These results suggest that cytochrome *c* failed to colocalize with mitochondria, which, in turn, suggests that a fraction of cytochrome *c* was released from the mitochondria in WDR79‐depleted cells (Fig. 5C). A similar result was also obtained in H1299 cells transfected with WDR79 siRNA (Fig. S3).

The Bcl‐2 family of proteins plays a pivotal role in the regulation of apoptosis by controlling mitochondrial permeability. The anti‐apoptotic protein Bcl‐2 located within the outer mitochondrial wall can inhibit cytochrome *c* release, whereas the pro‐apoptotic protein Bax promotes the release of cytochrome *c*. To further elucidate the mechanism of cytochrome *c* release, we examined the expression of Bcl‐2 and Bax in H1299 and A549 cells. After WDR79 was knocked down, the expression of Bcl‐2 significantly decreased, whereas Bax protein expression markedly increased, resulting in a low Bcl‐2/Bax ratio in both cell lines tested (Fig. 5D and E).

### Sustained suppression of WDR79 attenuates the tumorigenic potential of NSCLC *in vivo*

**Figure 6:**
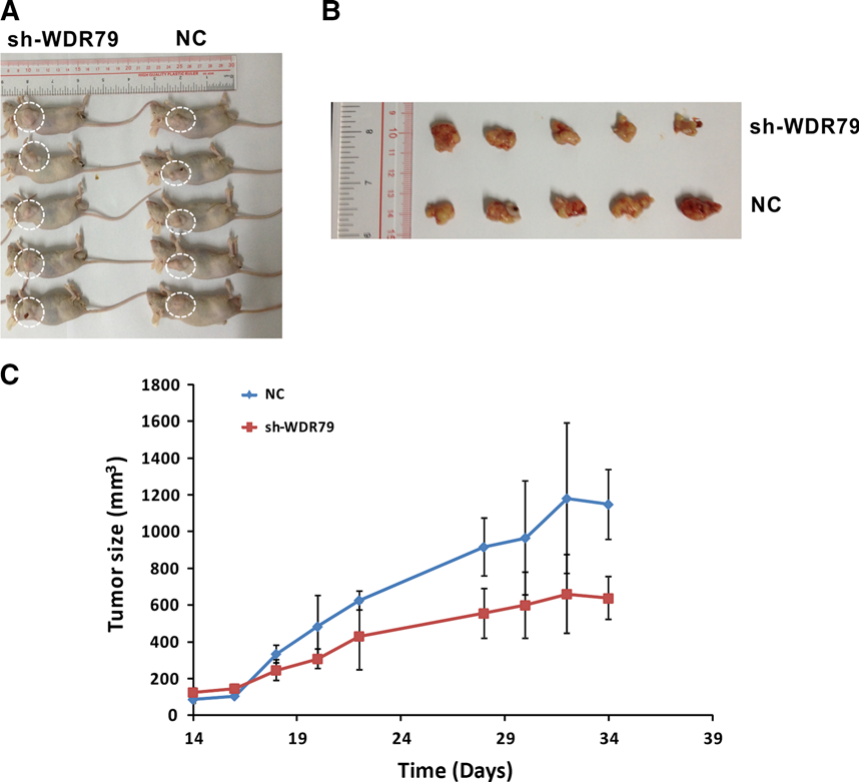
Inhibition of xenograft tumour growth *via* WDR79‐knockdown *in vivo*. A549 cells infected with WDR79 shRNA, control shRNA (NC) or blank were harvested, injected into mice, and allowed to grow for 34 days. (**A**) Representative images of human lung tumour xenografts in mice from the blank, NC and WDR79 shRNA groups. (**B**) Representative images of typical tumours from the three groups of mice. (**C**) Tumour volumes were measured on the indicated days using the methods described in the Materials and methods section.

To further investigate the role of WDR79 in NSCLC *in vivo*, we established NSCLC xenografts *via* subcutaneous injection of WDR79‐depleted A549 cells and control cells into the flanks of 8‐week‐old female BALB/c nude mice. After tumours developed into palpable masses approximately 2 weeks post‐inoculation in the WDR79‐knockdown and control groups, tumour dimensions were measured using callipers. Compared with the control group, the mean volume of the tumours derived from WDR79‐depleted cells was significantly smaller (Fig. 6A–C). These results suggest that the inhibition of WDR79 expression significantly suppresses NSCLC tumour growth *in vivo*.

## Discussion

WDR79 (also referred to as WRAP53, WRAP53β or TCAB1) is a WD‐repeat protein that participates in telomerase assembly and Cajal body formation 11, 12. This study is the first to report that WDR79 is overexpressed in clinical NSCLC tissues and cell lines and that it plays a critical role in NSCLC progression by regulating cell cycle and apoptosis.

Cell cycle is the main process driving cellular proliferation, which is induced by the sequential activation of protein kinase complexes comprising a cyclin and a CDK 24. In mammalian cells, cyclin D1/CDK4/6 complexes, together with cyclin E/CDK2 complexes, are activated in the G1 phase to control G1‐to‐S transition. Our studies indicated that WDR79‐knockdown in NSCLC cells caused cell cycle arrest at the G1 phase. The results obtained from evaluating the effects of WDR79 knockdown on the expression of G1‐related proteins in NSCLC cells revealed that cell cycle arrest was associated with the down‐regulation of cyclins (cyclin D1 and cyclin E) and CDKs (CDK2, CDK4 and CDK6). This decrease in cell cycle genes likely contributed to a decrease in the activation of G1‐related cyclins and CDK complexes, thus preventing cell cycle progression from the G1 to the S phase. Collectively, these results provide evidence that WDR79‐knockdown in NSCLC cells causes cell cycle arrest at the G0/G1 phase by affecting G1‐related cyclins and CDK complexes.

Previous studies have indicated that WDR79 is essential for Cajal body formation 25. Cajal bodies are dynamic nuclear organelles that have been implicated in various cellular processes 26. Defects in Cajal body formation are linked to impaired cell proliferation and apoptosis induction 27. For example, the depletion of the FLASH protein, a component of the Cajal body, inhibits cell growth and promotes apoptosis 28. Consistent with this, we found that the overexpression of WDR79 promoted NSCLC cell proliferation, whereas the down‐regulation of WDR79 resulted in the inhibition NSCLC cell proliferation. Further studies revealed that WDR79 knockdown in NSCLC cells induces apoptosis, as indicated by nuclear morphological changes and increased Annexin V and PI double staining.

Two distinct signalling pathways activate apoptosis, including the death receptor pathway (the extrinsic pathway) and the mitochondrial pathway (the intrinsic pathway). Cysteine proteases include initiator (upstream) and effector (downstream) caspases, which are involved in the two apoptotic pathways as regulators and activators. The extrinsic pathway is responsible for the activation of initiator caspase‐8 29, whereas the intrinsic pathway results in the activation of initiator caspase‐9 30. Both pathways converge at effector caspase‐3, which is cleaved and activated by initiator caspases, to trigger apoptosis. Our results revealed that WDR79 knockdown resulted in the activation of caspase‐9 and caspase‐3, rather than caspase‐8, suggesting that WDR79‐knockdown induces apoptosis in NSCLC cells *via* the mitochondrial pathway.

The mitochondrial pathway of apoptosis is characterized by cytochrome *c* release. The Bcl‐2 family proteins regulate apoptosis by controlling the mitochondrial membrane permeability. The anti‐apoptotic Bcl‐2 in the outer mitochondrial wall suppresses cytochrome *c* release, whereas pro‐apoptotic Bax mediates apoptosis by inducing the release of cytochrome *c* from the mitochondria. Previous studies have indicated that the ratio of Bcl‐2 to Bax determines the response to an apoptotic signal 31. The release of cytochrome *c* into the cytoplasm results in the activation of caspase‐9. Our results clearly demonstrated that WDR79 knockdown in NSCLC cells increased the level of Bax, while concomitantly decreasing Bcl‐2 levels, which lowered the ratio of Bcl‐2 to Bax. In coordination with the function of Bcl‐2 and Bax, the release of cytochrome *c* from the mitochondria and the activation of caspase‐9 were also observed after WDR79‐knockdown in NSCLC cells. Thus, our results provide evidence that WDR79 knockdown in NSCLC cells induces apoptosis *via* the mitochondrial pathway.

In summary, our results are the first to reveal that WDR79 is overexpressed in non‐small lung cancer tissues and cell lines. Moreover, WDR79 plays a key role in NSCLC tumorigenesis by regulating cell cycle progression and apoptosis. These findings provide a mechanistic basis for the further exploration of WDR79 as a diagnostic and therapeutic target for NSCLC.

## Conflicts of interest

The authors confirm that there are no conflicts of interest.

## Supporting information


**Figure S1** WDR79 expression is associated with cell proliferation.

**Figure S2** WDR79 affects cell cycle progression.

**Figure S3** WDR79 knockdown promotes the release of cytochrome c from mitochondria.
