# Supplementary figures and images for: Overexpression of WDR79 in non‐small cell lung cancer is linked to tumour progression
Source: J Cell Mol Med. 2016 Feb 5;20(4):698–709. doi: 10.1111/jcmm.12759 (PMC5125931; doi:10.1111/jcmm.12759)

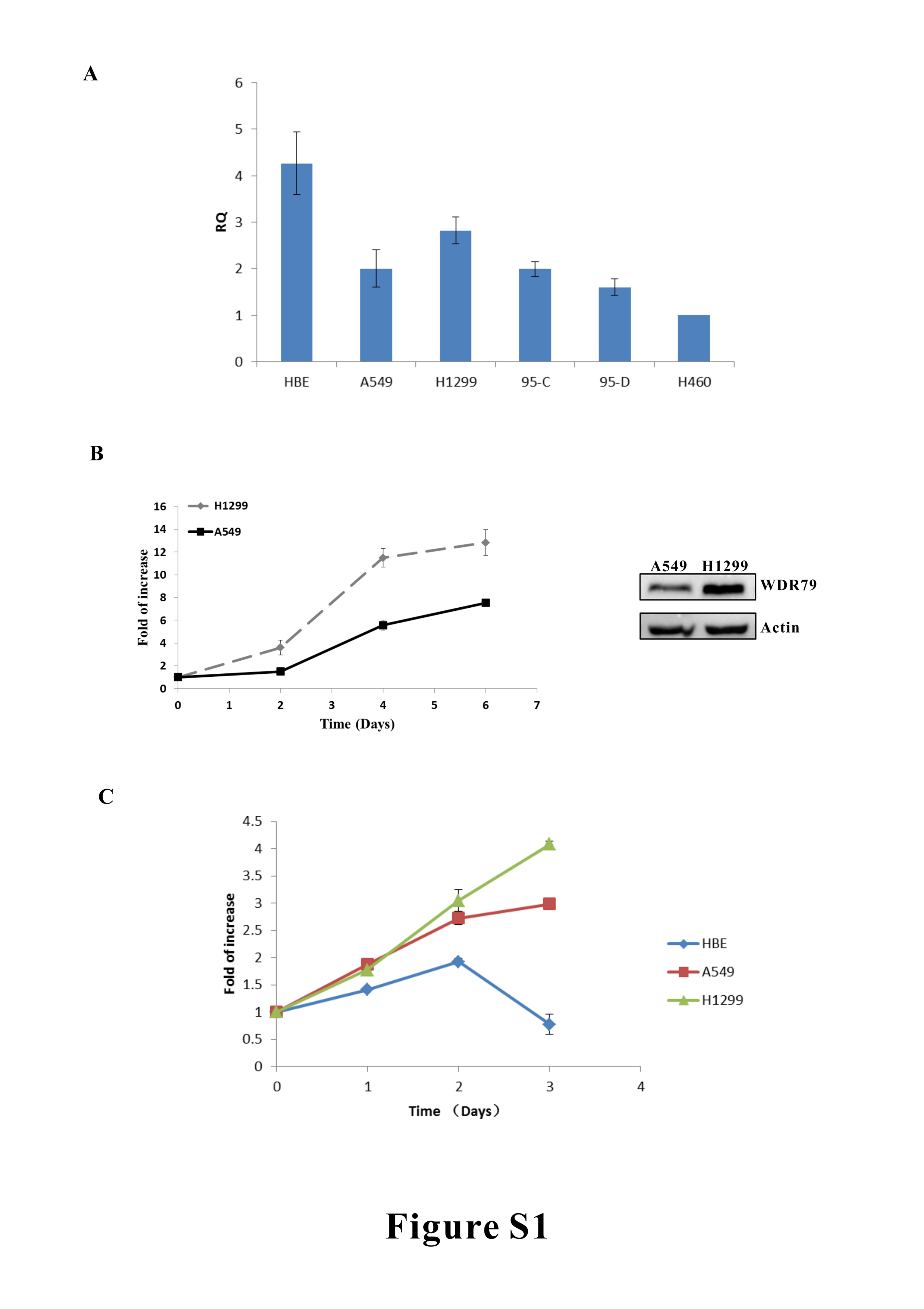

Supplement: Supplementary file 1 — Figure S1 WDR79 expression is associated with cell proliferation. [file JCMM-20-698-s001.tif]

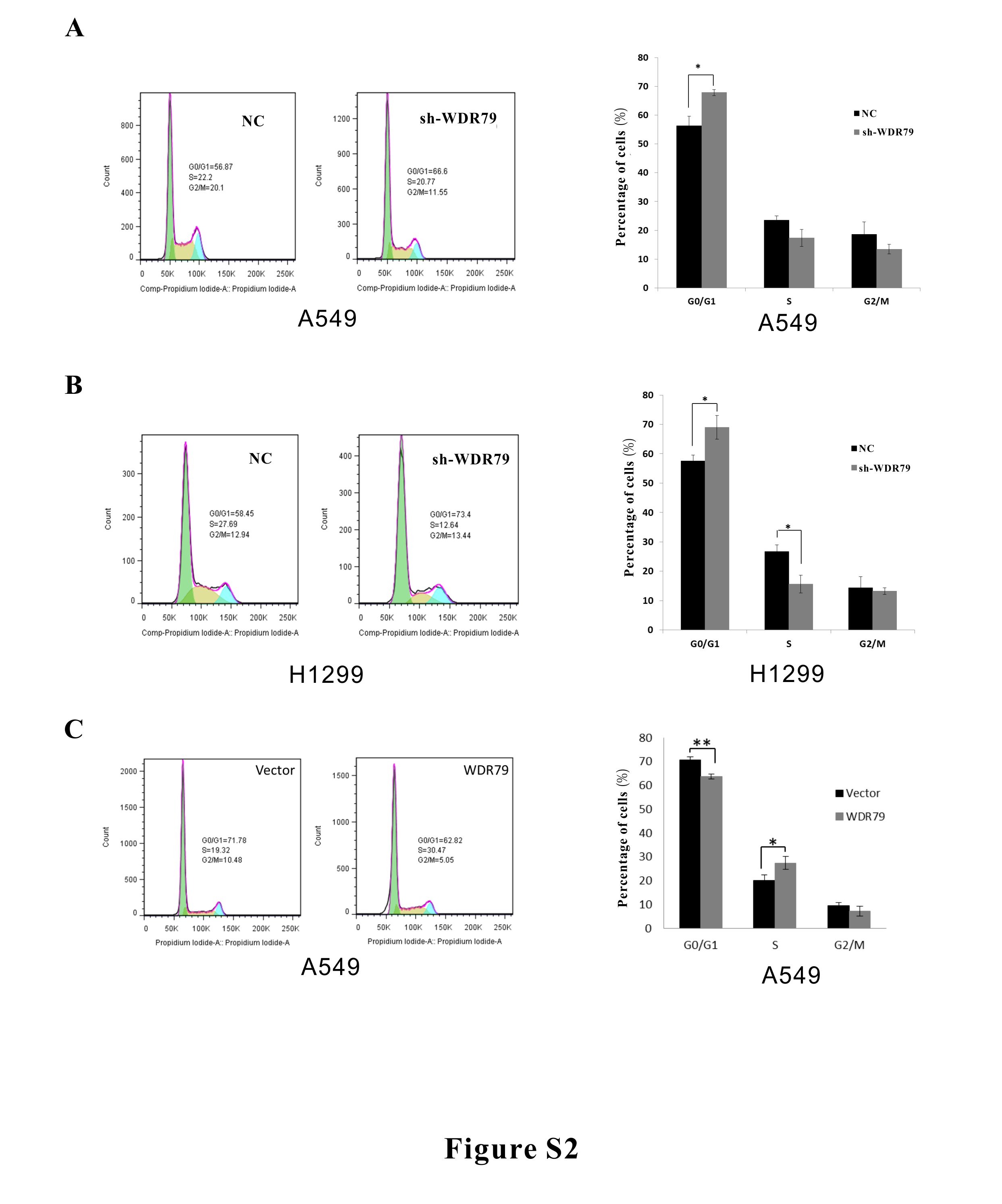

Supplement: Supplementary file 2 — Figure S2 WDR79 affects cell cycle progression. [file JCMM-20-698-s002.tif]

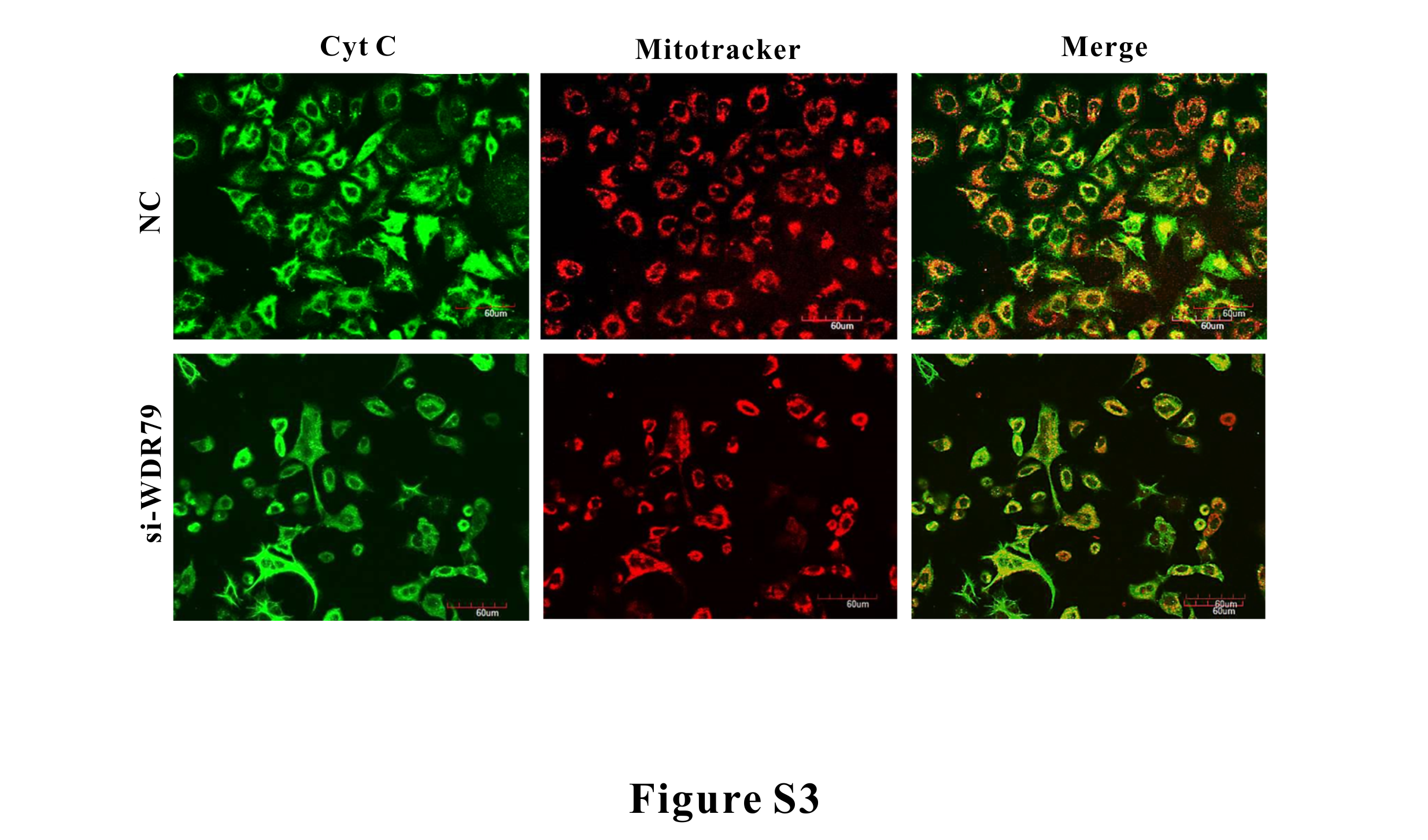

Supplement: Supplementary file 3 — Figure S3 WDR79 knockdown promotes the release of cytochrome c from mitochondria. [file JCMM-20-698-s003.tif]
