# Supplementary material for: Overexpression of WDR79 in non‐small cell lung cancer is linked to tumour progression
Source: J Cell Mol Med. 2016 Feb 5;20(4):698–709. doi: 10.1111/jcmm.12759 (PMC5125931; doi:10.1111/jcmm.12759)
Supplement: Supplementary file 4 [file JCMM-20-698-s004.doc]

**Supporting Information**

**Figure legends**

Figure S1 WDR79 expression is associated with cell proliferation. (A) Real-Time PCR were used to quantify WDR79 mRNA in different cell lines. (B) The proliferation of A549 and H1299 cells was determined via MTT assay at the indicated time points, and the expression of WDR79 protein was examined by western blotting using indicated antibodies. (C)The proliferation of HBE, A549 and H1299 cells was detected with MTT assay at the indicated time points.

Figure S2 WDR79 affects cell cycle progression. (A and B) After A549 (A) and H1299 (B) cells were infected with WDR79 shRNA or control shRNA, cells were subjected to cell cycle analysis. (C) After A549 cells were transfected with WDR79 plasmid or empty vector, cells were subjected to cell cycle analysis.

Figure S3 WDR79 knockdown promotes the release of cytochrome c from mitochondria. After H1299 cells were transfected with WDR79 siRNA or control siRNA, cells were fixed and then incubated with cytochrome *c* antibody, followed by staining with DyLight488-conjugated IgG for cytochrome *c* and Mitotracker for mitochondria.
